# Research on Optical Fiber Ring Resonator Q Value and Coupling Efficiency Optimization

**DOI:** 10.3390/mi14091680

**Published:** 2023-08-28

**Authors:** Shengkun Li, Xiaowen Tian, Sining Tian

**Affiliations:** School of Instrument and Electronics, North University of China, Taiyuan 030051, China

**Keywords:** resonant fiber gyroscope, fiber ring resonator, coupling efficiency, scale factor

## Abstract

The coupling efficiency of the fiber ring resonator has an important influence on the scale factor of the resonant fiber gyroscope. In order to improve the scale factor of the gyroscope, the coupling efficiency of the fiber ring resonator and its influential factors on the scale factor of the gyroscope are analyzed and tested. The results show that the coupling efficiency is affected by both the splitting ratio of the coupler and the loss in the cavity. When the coupling efficiency approaches 0.75 at the under-coupling state, the scaling factor of the gyroscope is the highest. This provides a theoretical reference and an experimental basis for the enhancement of the scaling factor of the resonant fiber gyroscope with the fiber ring resonator as the sensitive unit, providing options for multiple applications such as sea, land, sky and space.

## 1. Introduction

Inertial navigation is a self-service navigation system that does not need external information that radiates energy to the outside world. It is widely used in national defense [1], civil and commercial fields due to its advantages of long working hours, high stability and fast data update rate [2]. A gyroscope is the core component of an inertial navigation system [3], which is used to measure the angular velocity of the vector in the inertial space and the attitude of the vector [4]; the performance of the gyroscope plays a decisive role in the performance of the whole inertial navigation system [5]. Resonant gyroscopes use the resonant properties of vibration to measure rotation [6], and resonant fiber optic gyroscope (RFOG), a novel opto-electronic hybrid integrated sensor [7,8], measure the rotational angular velocity of the system by measuring the resonance frequency difference of clockwise (CW) and counterclockwise (CCW) light caused by the optical Sagnac effect. Due to its advantages of high precision, small size and no moving parts, the resonant optical gyroscope has drawn much attention [9]. Like ordinary mechanical gyroscopes, optical gyroscopes are also affected by coupling effects, temperature [10] and other external noise [11]. The optical gyroscope is favored in various application fields such as sea, land, sky and space because of its internal all-solid-state optical path, no rotor wear and no moving parts, which greatly improve the mechanical properties of inertial devices such as anti-vibration and shock resistance. Resonant fiber optic gyroscopes, as a kind of optical gyroscope, compared with interferometric fiber optic gyroscopes, only need to use a 2 × 2 coupler and pigtail fusion to form a resonant cavity, avoiding the temperature drift caused by the Shupe effect in the long optical fiber sensitive annulus At the same time, the miniaturization also shows its unique advantages and engineering application potential. A fiber ring resonator works as the core sensitive unit of the RFOG; it can be simply formed by splicing coupler pigtails. The low insertion loss allows light energy to be stored in the fiber core efficiently [12].

Although the fiber ring resonator (FRR) can improve the detection sensitivity of the gyroscope by obtaining a higher Q value, the effect of coupling efficiency on the gyroscopic scaling factor has not yet been fully analyzed theoretically and experimentally. Thus, in order to ensure the stability and accuracy of the gyroscope, it is necessary to reduce its temperature drift and coupling effects [13]. There are more research works on the direction of temperature drift compensation, and the combination of temperature compensation algorithms can effectively reduce the drift amount [14]. However, the cross-coupling effect [15,16] between multiple axes of different gyroscopes has some differences, and it is important to deeply explore and analyze its sources and mechanisms. The work in this paper focuses on the analysis of the coupling states of FRR, which has three coupling states, namely, the states of under-coupling, over-coupling and critical coupling [17]. Experiments are conducted under different coupling states to investigate in depth the effects of different coupling states on the accuracy of gyroscopes and to provide an experimental basis for the subsequent related calibration and compensation.

The influence factors of the three coupled states of the fiber ring resonator are studied in this paper. The influence of the coupled state on the detection accuracy of the resonant optical gyroscope is studied through theoretical analysis, modeling simulation and experimental testing. The experimental results show that the coupling efficiency of the resonant cavity is mainly influenced by the cavity loss and the split ratio of coupling. When the coupling efficiency approaches 0.75, the gyroscope has the highest scale factor and is in the under-coupling state. The cavity length is 3 m, and, when the split ratio is 2/98, a resonator with Q value of 2.3 × 10^8^ and coupling efficiency of 0.7334 is obtained, with the highest top scale factor of 118.2876 mV/°/s.

## 2. The Analysis of Principle

### 2.1. Coupling Efficiency (ρ)

As the core component of the fiber ring resonator, the fiber coupler uses the evanescent field coupling of the light waves to realize the splitting and combining between the adjacent fibers [18].

**Figure 1:**
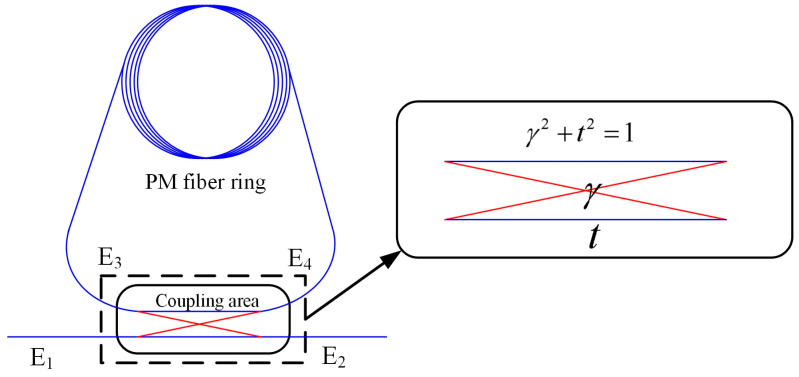
The structure of FRR.

Figure 1 shows the fiber ring resonator. E_1_, E_2_, E_3_ and E_4_ are the intensity of the light field propagating in ports 1, 2, 3 and 4. Light enters the fiber coupler from port 1, and part of it is output from port 2 directly. The other part of the light is coupled into the cavity via port 4 and continues to propagate through the coupling area for one more revolution before entering the coupler through port 3 again. The light entering port 3 continues to propagate through port 4, while the other portion enters the coupling region to exit port 2. E_1_, E_2_, E_3_ and E_4_ satisfy the following relationship [19]:

(1) $$E_{2}=\gamma E_{1}+jtE_{3}$$

E_4_ = *γ*E_3_ + *jt*E_1_ (2)

*γ*^2^ + *t*^2^ = 1 (3)

*γ* and *t* are the coupling and transmission coefficients, respectively, and *j* is the imaginary unit.

The loop light intensity transfer factor *τ* and transmission phase shift *φ* are used to describe the propagation of light in the fiber ring resonator:

E_2_ = *τ* exp(*iφ*)E_4_ (4)

where *φ* = *κL*, *κ* = 2*πn*/*λ*, *n* is the effective refractive index of the fiber, *λ* is the wavelength of the incident light in vacuum and *L* is the cavity length of the fiber ring resonator.

From (1)–(4), the relationship between E_1_ and E_2_ can be expressed:

(5) $$\frac{E_{2}}{E_{1}}=\exp\left[i\left(\pi +\varphi \right)\right]\frac{\tau -\gamma \exp\left(-i\varphi \right)}{1-\gamma \tau \exp\left(-i\varphi \right)}$$

The transmission light can be obtained by the square of Formula (5):

(6) $$T=\left|\frac{E_{2}}{E_{1}}\right|=\frac{\tau^{2}-2\gamma \tau \cos\varphi +\gamma^{2}}{1-2\gamma \tau \cos\varphi +\gamma^{2}\tau^{2}}$$

Based on the above calculations, the coupling efficiency can be calculated as follows [9]:

(7) $$\rho =1-{\left(\frac{\gamma -\tau }{1-\gamma \tau }\right)}^{2}$$

**Figure 2:**
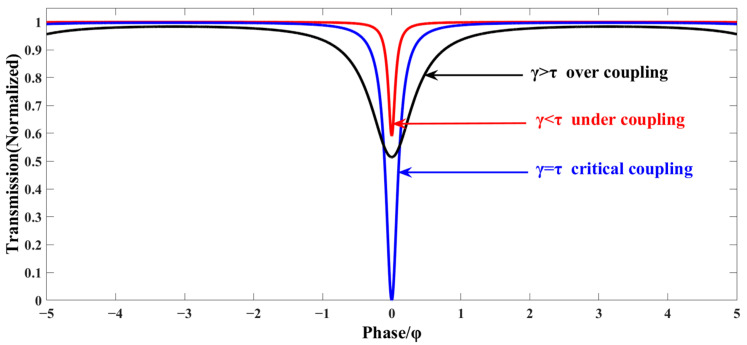
Simulation of coupling state.

The coupling state is shown in Figure 2. When *γ* = *τ* and *T* = 0, the output light intensity of E_2_ is zero. The optical energy loss and replenishment in the resonator reach dynamic balance. The FRR is in the critical coupling state and the coupling efficiency is the highest. In this case, the light coupled to the FRR by E_1_ is equal to the light loss in the cavity (including the insertion loss, the transmission loss in the fiber loop and the loss in the splice). When *γ* > *τ*, the optical energy in the cavity is replenished more than the loss, the cavity is in an over-coupled state. Large full width at half maximum (FWHM) results in low Q factor. When *γ* < *τ*, the cavity light energy is less than the loss of replenishment, the resonator is in a state of under-coupling, the full width at half maximum is narrow and the quality factor is high.

**Figure 3:**
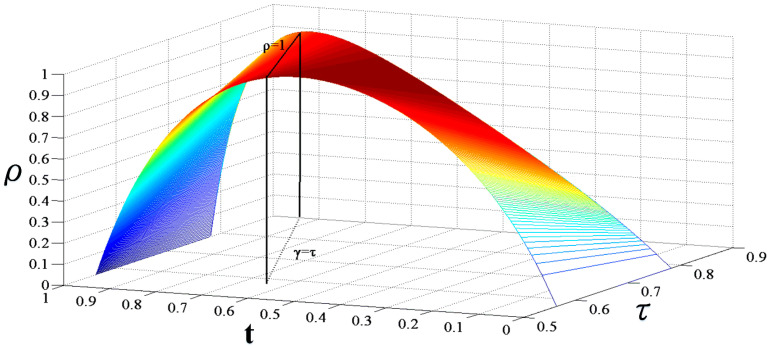
The influence of splitting ratio and loss on coupling efficiency.

The relationship between the splitting ratio and the loss on the resonance depth is shown in Figure 3. The coupling state of the resonator is affected by the different matching relationship between the cavity loss and the splitting ratio of the coupler. When *γ* = *τ*, the cavity coupling efficiency is the highest.

### 2.2. The Scale Factor of the Gyroscope

In the RFOG signal detection system, the triangular wave phase modulation technology is used to modulate the light emitted by the laser. If the output frequency of the modulated laser turns from *f*_0_ to *f*_0_ ± Δ*f*, the gyroscope output [17,19] is

(8) $${Ι}_{out}={Ι}_{in}\left(\frac{r^{2}+a^{2}-2ra\cos\left(\frac{2\pi \left(f_{0}+2\Delta f\right)}{FSR}\right)}{1+r^{2}a^{2}-2ra\cos\left(\frac{2\pi \left(f_{0}+2\Delta f\right)}{FSR}\right)}-\frac{r^{2}+a^{2}-2ra\cos\left(\frac{2\pi \left(f_{0}-2\Delta f\right)}{FSR}\right)}{1+r^{2}a^{2}-2ra\cos\left(\frac{2\pi \left(f_{0}-2\Delta f\right)}{FSR}\right)}\right)$$

We demodulate the slope of the curve under the condition of a certain free spectral range (*FSR*) of the resonance spectrum, which is the effect of fiber ring resonator coupling efficiency on gyroscope scale factor:

(9) $$\kappa =\frac{d{Ι}_{out}}{\Delta f}{|}_{\Delta f=0}$$

From Equations (8) and (9), it can be known that, when the maximum value is taken, the following relation is obtained:

(10) $$r=\frac{2\tau +1}{\tau +2}$$

In this case, the coupling efficiency *h* = 0.75, that is, when *r* and *τ* satisfy the relationship shown in Formula (10), the slope of the RFOG demodulation curve is the largest, which means that the gyroscope has the highest scale factor. In this case, the fiber ring resonator is in an under-coupled state and the coupling efficiency is 0.75.

The proper match between splitting ratio and the cavity loss in the coupler constitutes a resonant cavity with a coupling efficiency of 0.75. Losses in FRR include insertion loss, transmission loss in fiber and splice loss. Insertion loss and fiber transmission loss (0.15 dB/km) are very low compared to splice loss, so splice loss constitutes the major loss in the cavity. The splice loss was tested in the experiment.

## 3. The Experimental Test

### 3.1. Melting Point Loss Experiment

**Figure 4:**
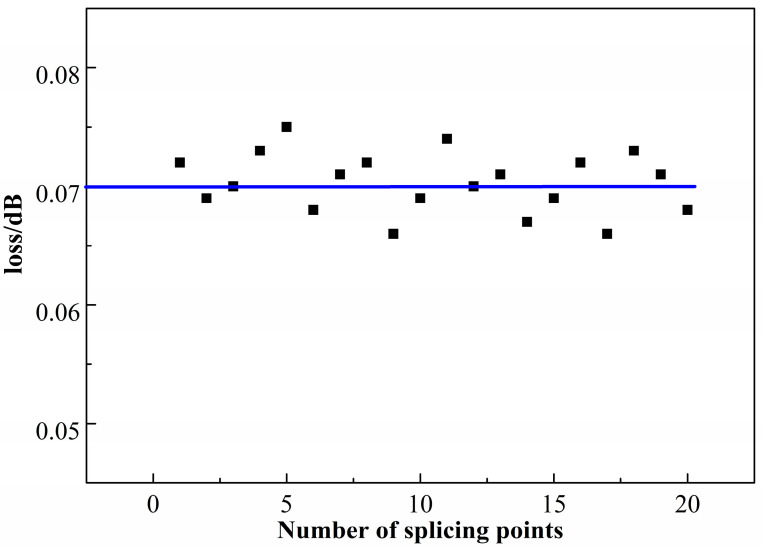
Loss in splicing points.

In order to obtain the wastage of the melting point, we cut off a polarization-maintaining optical fiber, and then welded it in the same welding environment and parameters. Optical fiber lasers were connected at one end, and the other end accessed ab optical power meter and read the light power before and after welding. We tested many times to obtain the average; the results are shown in Figure 4. The loss caused by each fusion point was about 0.07 dB, and the loss caused by the two melting points was about 0.14 dB.

### 3.2. Optical Fiber Ring Resonator Test

**Figure 5:**
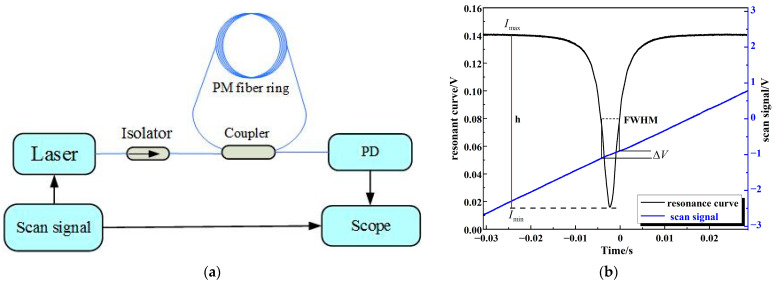
(**a**) Resonant spectrum test system, (**b**) coupling efficiency and Q factor calculation.

In the resonant spectrum test system, as shown in Figure 5a, a narrow line-width laser (100 Hz) with a wavelength of 1550 nm was adopted as a laser light source, the frequency sweep coefficient was 15 MHz/V, the signal generator generated a triangle wave as the laser scanning signal and the resonance line, after photoelectric detection in photoelectric conversion, showed the form of the voltage signal on the oscilloscope. The definition of coupling efficiency is $h=\frac{I_{\max}-I_{\min}}{I_{\max}}$, where *I*_max_ is the maximum value of the resonance spectrum, *I*_min_ is the minimum value of the resonance spectrum and *I*_max_ − *I*_min_ is the amplitude range of the resonance spectrum line. The full width at half maximum (FWHM) method was adopted to calculate the Q value. The FRR resonance state was stimulated under the frequency sweep mode, sampling resonance on the scope and scanning the data of voltage. Origin software was used to calculate the scanning signal voltage difference ΔV corresponding to the FWHM of the resonance line. The laser scanning coefficient was 15 MHz/V and the corresponding frequency of light wave at the output center wavelength of 1550 nm was *f*_0_ = 193 THz. The Q value of the resonant cavity can be calculated by the formula $Q=\frac{f_{0}}{\mathrm{FWHM}}=\frac{f_{0}}{\Delta V\times 15\;\mathrm{MHz}/V}$ [20]. The coupling efficiency and the Q value of the FRR are shown in Figure 5b.

**Figure 6:**
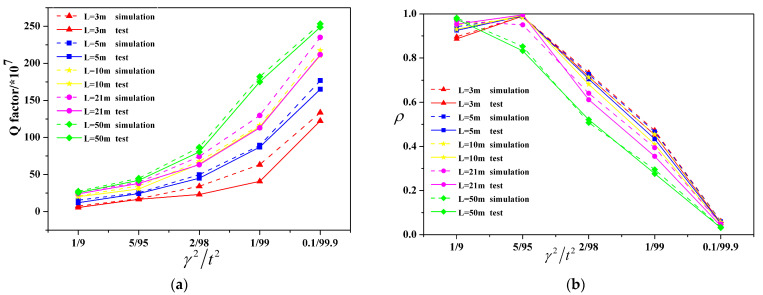
(**a**) Comparison of measured and simulated Q values for different lengths and splitting ratios, (**b**) comparison of measured and simulated coupling efficiency for different lengths and splitting ratios.

In the experiment, five kinds of optical split ratio couplers (1/9, 5/95, 2/98, 1/99 and 0.1/99.9) were combined into fiber ring resonators with five cavity lengths (3 m, 5 m, 10 m, 21 m and 50 m), and the system shown in Figure 5 was used for testing and comparison with the simulation results. The results are shown in Figure 6a,b. The Q value of the resonant cavity shows an increasing trend with decrease in the splitting ratio and increase in the cavity length. The coupling efficiency is influenced by the splitting ratio of couplers and the loss in the cavity, showing a trend of an initial rise and then a decrease. This is because a high splitting ratio means energy supply is greater than the loss in the cavity, which is in the over-coupling state; additionally, the coupling efficiency and Q value are low. With further decrease in the splitting ratio, the complementary and dissipation of light energy in the cavity gradually reached dynamic equilibrium. The coupling efficiency and the Q value were gradually increased, approaching the critical coupling state. Further reduction in the splitting ratio caused the light energy supplement in the cavity to be less than the loss. In the under-coupled state, the Q value gradually increased, and the coupling efficiency showed a downward trend. The declining green curve in Figure 6b is due to the longer length of the cavity causing a bigger transmission loss and to melt contact loss accumulation making the optical energy loss in the cavity greater than the optical energy supplement to the resonant cavity under each splitting ratio; therefore, this was in the under-coupling state. The loss cavity length was 3 m. The Q value of the resonant cavity was 2.3 × 10^8^ while the splitting ratio of the resonator was 2/98, and the coupling efficiency was 0.7334. Therefore, in the case where the optical fiber fusion point loss is known, by adopting the appropriate splitting ratio tie-in cavity length, the highest Q value and coupling efficiency of the optical FRR, i.e., 0.75, can be theoretically obtained.

### 3.3. Resonance Fiber Optic Gyroscope Index Test

**Figure 7:**
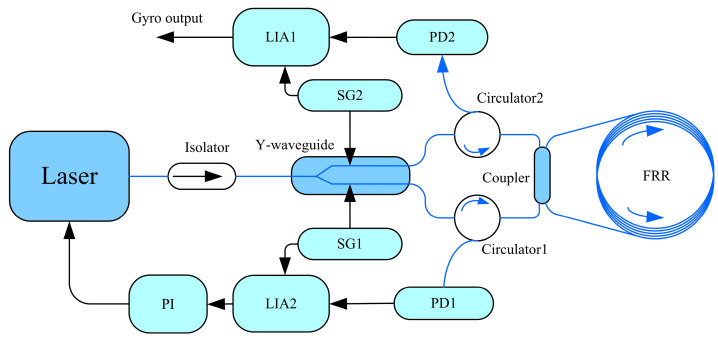
Schematic diagram of the ROG.

The above FRRs with different coupling efficiencies were tested in the RFOG system shown in Figure 7. Laser was emitted with stable power and wavelength of 1550 nm, and it was divided into two identical beams of light through a Y-waveguide. The two-phase modulation arms in the Y-waveguide phase modulator were used for the CW and CCW directions of optical signal modulation, in which the modulation frequencies were 335 kHz and 550 kHz, respectively. The two channels of light are resonant after the coupler; they enter the resonator cavity, pass through the circulator, are converted into an electrical signal in the photodetector and are output to the lock-in amplifier to be demodulated. In the CCW road, the output frequency of the laser is locked in as the resonant frequency of the fiber ring resonator through proportional Integration controller; the CW road is gyroscope output.

**Figure 8:**
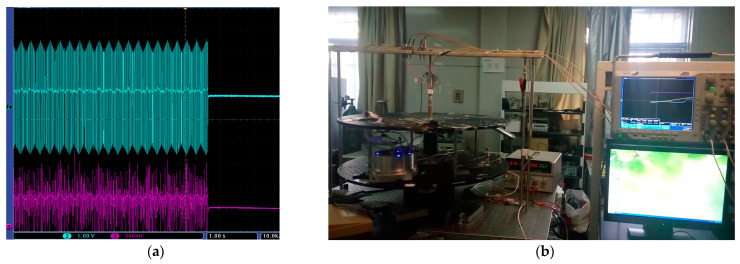
(**a**) Laser frequency locking; (**b**) Rotation test.

As shown in Figure 8, the prototype was fixed on the rotary table at room temperature. The frequency locking of the laser was realized by sending the frequency locking control command from the host computer, as shown in Figure 8a, to control the angular velocity output of the high-precision rotary table and to calibrate its scaling factor by using the step signals corresponding to the individual angular velocities. The output rotational angular velocity of the rotary table was controlled by the upper computer.

**Figure 9:**
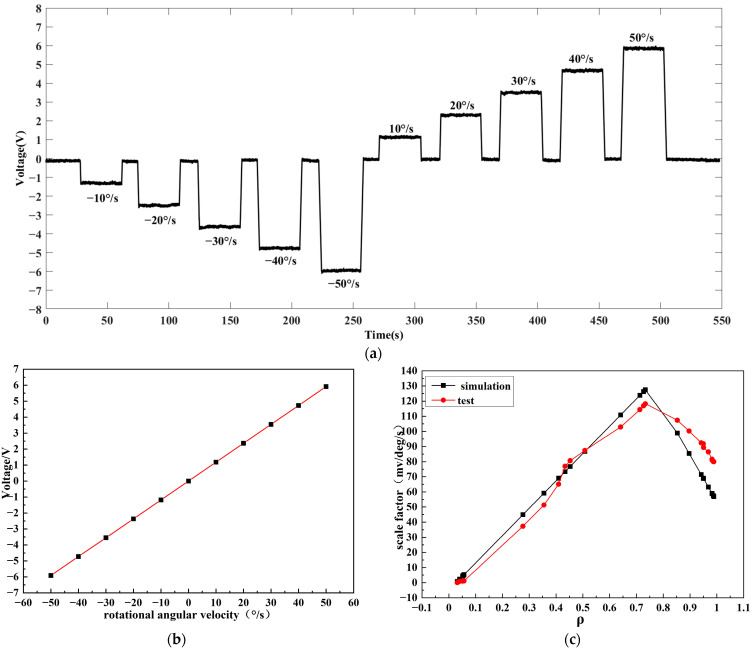
(**a**) Gyroscope signal with different rotational angular velocities; (**b**) fitting analysis of stair effects; (**c**) comparison of measured and simulated values of ROG sensitivity for the different coupling efficiency values.

The system was fixed on the high precision turntable with the angular velocity set at about 10°/s, 20°/s, 30°/s, 40°/s and 50°/s for rotation tests. We performed rotation at each angular velocity three times, taking the average of the output voltage as rotation output signal. The results in Figure 9a show the least-square linear fitting of the output voltage corresponding to each angular velocity, used to obtain the curve of angular velocity and the output voltage as shown in Figure 9b. The calculated gyroscope scale factor was 118.2876 mV/°/s.

For the data in Figure 9c, the FRR with 25 parameters was tested in the gyroscope system, comparing the measured value with the theoretical calculation; the actual measurement values were basically the same as those from the theoretical calculation. With the increase in coupling efficiency, the scale factor of the gyroscope first increases and then decreases. For the scale factors between 0.641 and 0.8966, all values exceeded 100 mV/°/s, with the highest point being 118.2876 mV/°/s, corresponding to a coupling efficiency of about 0.7334. Therefore, under the current test conditions, it is necessary to control the coupling efficiency to values between 0.641 and 0.8966 to achieve a higher scale factor. There is a certain deviation between the measured value and the theoretical calculation value because the FRR has a certain error in the light ratio and the calculation error of the melting point loss.

## 4. Conclusions

In this paper, the influencing factors of coupling efficiency on FRR are modeled, and the influence of coupling efficiency on the scale factor of a resonant optical gyroscope is analyzed in detail. The Q factor and coupling efficiency test system of the FRR and the resonant fiber gyroscope system are built, and the scale factor of the gyroscope is tested with a high-precision rotating platform. Both theoretical analysis and experimental results show that the coupling efficiency of the resonant cavity is influenced by cavity loss and the splitting ratio of coupling. The scale factor of the gyroscope is the highest when the coupling efficiency approaches 0.75 in the under-coupling state. The cavity length is 3 m, and, when the splitting ratio is 2/98, a resonator with a Q value of 2.3 × 10^8^ and a coupling efficiency of 0.7334 is obtained. In addition, the top scale factor is the highest, which is 118.2876 mV/°/s. This paper provides theoretical and experimental support for improving the scale factor of an RFOG with a fiber ring resonator as the sensitive element. It is of great significance to improve the detection accuracy of the gyroscope’s rotational angular velocity in the carrier, to enhance the initial alignment accuracy of the inertial navigation system, to obtain the carrier orientation and attitude data more accurately for the control system and to improve the accuracy and stability of the carrier’s running attitude.

## Data Availability

Not applicable.
